# Relationship between Lichen Planus and Helicobacter pylori Infection

**DOI:** 10.5681/joddd.2010.005

**Published:** 2010-03-14

**Authors:** Ali Taghavi Zenouz, Masoumeh Mehdipour, Mohammad Jafari Heydarlou, Narges Gholizadeh

**Affiliations:** ^1^ Assistant Professor, Department of Oral Medicine, Faculty of Dentistry, Tabriz University of Medical Sciences, Tabriz, Iran; ^2^ Assistant Professor, Department of Oral Medicine, Faculty of Dentistry, Uremia University of Medical Sciences, Uremia, Iran

**Keywords:** Helicobacter pylori, lichen planus, urea breathing test

## Abstract

**Background and aims:**

Lichen planus (LP) is a relatively common, chronic dermato-mucosal disease that often affects the oral mucosa. Among bacterial infections affecting LP, Helicobacter pylori has recently been proposed as an important etiologic factor. The present study was designed to evaluate the association of LP and H. pylori infection.

**Materials and methods:**

This study included 30 patients with skin LP, 30 patients with oral LP and 30 healthy individuals without LP as control group. Patients and control group were selected from those referred to a dental and a dermatology clinic. Urea breathing test (UBT) was performed for all subjects. Descriptive statistic (frequency and percentage) were applied and chi-square test was employed to compare mean differences, using SPSS 13.0 computer software.

**Results:**

UBT test were positive in 24 patients (80%) in oral LP group, 22 patients (73.3%) in skin LP group, and 20 individuals (66.7%) in the control group. No significant differences were found in the positive test results between the three groups (P = 0.50).

**Conclusion:**

In this study, no significant association was found between LP and H. Pylori infection.

## Introduction

Lichen planus (LP) is a relatively common, chronic dermato-mucosal disease that often affects the oral mucosa.^1^ LP has a worldwide distribution with no overt racial predisposition.^2^ Prevalence rates of oral lichen planus (OLP) vary from 0.5 to 2.2%. Females are more commonly affected than males. The mean age at the time of diagnosis is approximately 55 years and the lesions are considered premalignant.^3^ The disease has rarely been reported in children.^1^ LP can affect any part of the skin or mucosa but is most commonly seen in flexor surfaces of the wrists, the back and the ankles.^2^ Mucous membrane lesions are very common, occurring in 30–70% of cases. In 15% of the cases the lesions are limited to the oral mucosa.^2^

Although the etiology and pathogenesis of LP is not fully understood, different causes including genetic susceptibility, stress and anxiety, depression, hypersensitivity to drugs, metals and vaccinations, diabetes, hepatitis C, trauma, autoimmune diseases, and bacterial and viral infections may act as risk factors for LP.^2 - 4^ Among bacterial infections that may initiate LP, *Helicobacter pylori* infection has received attention as an important etiologic factor.^4^ There are reports that indicate *H. pylori* may be the cause of peptic ulcers as well as non-gastrointestinal diseases such as psoriasis.^5^

In a recent study based on urea breathing test (UBT), *H. pylori* was seen to be significantly higher in patients with LP compared to individuals with other skin diseases.^4^ However, a previous study employing the same method had failed to show such a significant difference.^5^ In another study evaluating serum IgG, although *H. pylori* infection was present in 66% of LP patients, no significant difference was seen in comparison with otherwise healthy controls.^6^

Considering the controversy over the association of LP with *H. pylori* infection in the limited available literature, the aim of the present study was to separately evaluate the association of oral and skin lichen planus with *H. pylori* infection using UBT method.

## Materials and methods

The study subjects were selected from those referred to the Department of Oral Medicine at Tabriz University of Medical Sciences Faculty of Dentistry or to the Dermatology Clinic at Sina Hospital.

Patients with history of using antibiotics, H_2_ inhibitor agents or Omeperazole during the past 15 days, and bismuth during the past 1 month, and patients with concurrent oral and skin lichen planus lesions were excluded from this study.

Sampling was done by simple non-random method. Subjects included 30 patients with skin lichen planus, 30 patients with oral lichen planus and 30 healthy individuals without lichen planus as control group.

After taking medical history, a complete examination, filling out an initial checklist, and performing biopsies, UBT was performed for all the groups using Helkit IRO3 equipment (ISODIAGNOSTIKA, Edmonton, Alberta, Canada).

Descriptive statistic (frequency and percentage) were applied to data and chi-square test was employed to compare mean differences, using SPSS 14.0 computer software.

## Results

Mean age of patients was 40 ± 12 years in the OLP group, 39 ± 9 years in skin LP group, and 37 ± 12 years in the control group. No statistically significant differences were found regarding age between the groups (P = 0.44, F _(2, 87)_ = 0.81). The OLP group consisted of 15 males (50%) and 15 females(50%); the skin LP group consisted of 17 males (56.7%) and 13 females (43.3%) and the control group consisted of 19 male s(63.3%) and 11 (36.7%)females. No statistically significant differences were found between the groups regarding gender distribution (P = 0.58, df =2, χ^ 2^ = 1.08).

**Figure 1:**
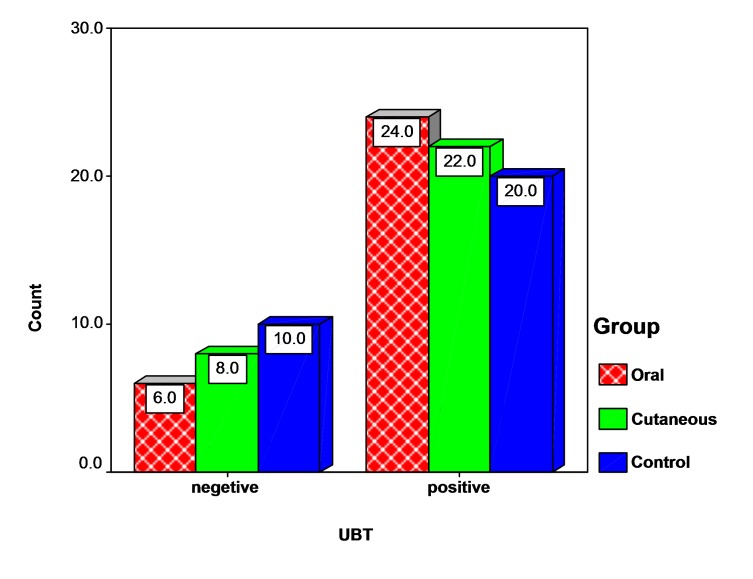
Distribution of UBT results in the case and control groups.

UBT test was positive in 24 patients (80%) in OLP group, in 22 patients (73.3%) in skin LP group, and in 20 individuals (66.7%) in the control group (Figure 1). The means of UBT titers were 14.3 ± 12.18 in the OLP group, 11.53 ± 10.13 in the skin LP group, and 12.22 ± 13.37 in the control group. No significant differences were found in the positive test results between the three groups (P = 0.50, df = 2, χ^ 2^ = 1.36). Mean UBT titers between the groups also did not show any significant differences (P = 0.65, F _(2, 87)_ = 0.43).

## Discussion

*H. pylori* species has been reported to have different prevalence rates in different countries, depending on oral hygiene status of the area. In developing countries, 80% of the populations are infected under the age of 20. This microorganism is rarely seen in children in the USA. The prevalence of *H. pylori* is approximately 30% in the USA.^7^

Invasive and non-invasive tests are available for identification of *H. pylori*. Invasive tests require upper gastrointestinal endoscopy, which is not often performed in the initial management of young patients. Noninvasive *H. pylori* testing is the common method if gastric cancer does not need to be excluded by endoscopy. The most consistent and accurate test is UBT, which bears 100% sensitivity and 95% specificity. The stool antigen test is more convenient and potentially less expensive than the UBT. The simplest tests for ascertaining *H. pylori* status are serologic assays measuring specific IgG level in serum. This test is not used to monitor treatment success, as the gradual drop in titer of H. Pylori-specific antibodies is too slow to be of practical use.

In the present study, approximately 66.7% of control group were infected with *H. pylori*. However, since a positive UBT result indicates active infection, these results should be considered more carefully. With regard to the fact that *H. pylori* is a normal micro-flora of the oral mucosa and not the skin, we separated OLP patients from skin LP cases in the present study. Patients with both oral and skin LP were excluded from the study, in order to allow for a separate evaluation of *H. pylori* interaction with each type of lichen planus.

Previous studies have failed to evaluate the association of *H. pylori* and each type of lichen planus separately. Vainio et al,^6^ studying the association of peptic ulcer and *H. pylori* in patients with LP and other skin disorders, could not find a significantly higher level of *H. pylori* infection in the test group including LP patients. This study could not attribute any etiologic role for *H. pylori* in LP.

Riggio et al^8^ evaluated *H. pylori* in recurrent aphthous and OLP by polymerase chain reaction (PCR) in 28 patients with aphthous, 20 patients with OLP, and 13 healthy individuals as controls. Three patients with aphthous were positive for *H. pylori*; however, this study could not attribute any etiologic role for *H. pylori* in OLP.

Shimoyama et al^9^ studied the association of *H. pylori* and oral mucosal ulcerative disorder: 12 cases of recurrent aphthous stomatitis (RAS), 3 cases of erosive LP and 7 cases with herpes simplex virus (HSV). Serum IgG antibodies were examined against *H. pylori* in all cases and samples were taken from the oral lesions, and cultured. In the latter study, all the RAS and LP cases were culture-negative for *H. pylori*, while two cases of HSV were positive. The two culture-positive cases were also seropositive for the *H. pylori* antigen. No relationship was found between *H. pylori* and the various oral ulcers based on serum IgG levels against *H. pylori*.

In a study on 61 patients with LP, 84 patients with psoriasis, and 58 healthy controls,^5^ 75.4% showed positive UBT with a mean titer of 35.46 and 74.1% of their controls showed positive UBT with a mean titer of 21.50, which shows no statistically significant differences between the patients and their controls. All the patients with OLP took therapeutic regimes for elimination of *H. pylori* infection. In 3 subjects oral lesions extended, in 4 subjects no changes were observed and in 3 subjects lesions showed relative remission. This study also could not attribute any etiologic role for *H. pylori* in LP. In another study, *H. pylori* was evaluated in 43 patients with RAS and 44 non-RAS control group using UBT.^10^ 16 instances in the RAS patients (37.2%) and 14 individuals in the control group (31.8%) showed positive UBT. The difference was not considered statistically significant (P = 0.597).

However, the results of a recent study showed that UBT was positive in 82.5% of LP patients, while in the control group (with other skin disease) 61.25% had positive UBT.^4^ The mean titer was 202.2 in patients with LP and 105.1 in the control group. The difference between the patients with LP and the control group were statistically significant (P = 0.0001) and the pathogenic role of *H. pylori* in LP was considered to be highly probable.^4^ The latter study was done on Iranian subjects, and our study was also conducted on Iranian subjects. The prevalence of *H. pylori* is relatively high in Iran. Despite the association of *H. pylori* and LP shown in the mentioned study,^4^ we could not demonstrate such a relationship. The prevalence of positive results in the control group in our study was 66.7% whereas the prevalence of positive results in the controls in the latter study was 61.25%. Most subjects (24 patients) in our study had ulcerative LP, whereas in that study most subjects had skin LP. We evaluated oral and skin LP separately. The differences between the results of the present study and the latter could be attributed to such differences in study design. Further studies are suggested with elimination treatment of *H. pylori* in the UBT-positive case group and comparison of the mean differences with the control group.

## Conclusion

According to results, no significant associations were found between OLP and *H. pylori* in the study groups.
